# The impact of ageing mechanisms on musculoskeletal system diseases in the elderly

**DOI:** 10.3389/fimmu.2024.1405621

**Published:** 2024-05-07

**Authors:** Yijin Cai, Zhongyu Han, Hong Cheng, Hongpeng Li, Ke Wang, Jia Chen, Zhi-Xiang Liu, Yulong Xie, Yumeng Lin, Shuwei Zhou, Siyu Wang, Xiao Zhou, Song Jin

**Affiliations:** ^1^ School of Medical and Life Sciences, Chengdu University of Traditional Chinese Medicine, Chengdu, China; ^2^ School of Automation Engineering, University of Electronic Science and Technology, Chengdu, China; ^3^ Eye School of Chengdu University of Traditional Chinese Medicine, Chengdu, China; ^4^ School of Health Preservation and Rehabilitation, Chengdu University of Traditional Chinese Medicine, Chengdu, China; ^5^ Jiangsu Key Laboratory of Molecular and Functional Imaging, Department of Radiology, Zhongda Hospital, School of Medicine, Southeast University, Nanjing, China; ^6^ Department of Gastroenterology, The First Hospital of Hunan University of Chinese Medicine, Changsha, China; ^7^ Second Clinical Medical College, Heilongjiang University of Chinese Medicine, Heilongjiang, China; ^8^ Department of Rehabilitation, Hospital of Chengdu University of Traditional Chinese Medicine, Chengdu, China

**Keywords:** ageing, cell senescence, SASP, osteoarthritis, osteoporosis, sarcopenia

## Abstract

Ageing is an inevitable process that affects various tissues and organs of the human body, leading to a series of physiological and pathological changes. Mechanisms such as telomere depletion, stem cell depletion, macrophage dysfunction, and cellular senescence gradually manifest in the body, significantly increasing the incidence of diseases in elderly individuals. These mechanisms interact with each other, profoundly impacting the quality of life of older adults. As the ageing population continues to grow, the burden on the public health system is expected to intensify. Globally, the prevalence of musculoskeletal system diseases in elderly individuals is increasing, resulting in reduced limb mobility and prolonged suffering. This review aims to elucidate the mechanisms of ageing and their interplay while exploring their impact on diseases such as osteoarthritis, osteoporosis, and sarcopenia. By delving into the mechanisms of ageing, further research can be conducted to prevent and mitigate its effects, with the ultimate goal of alleviating the suffering of elderly patients in the future.

## 1 Introduction

**Figure 1:**
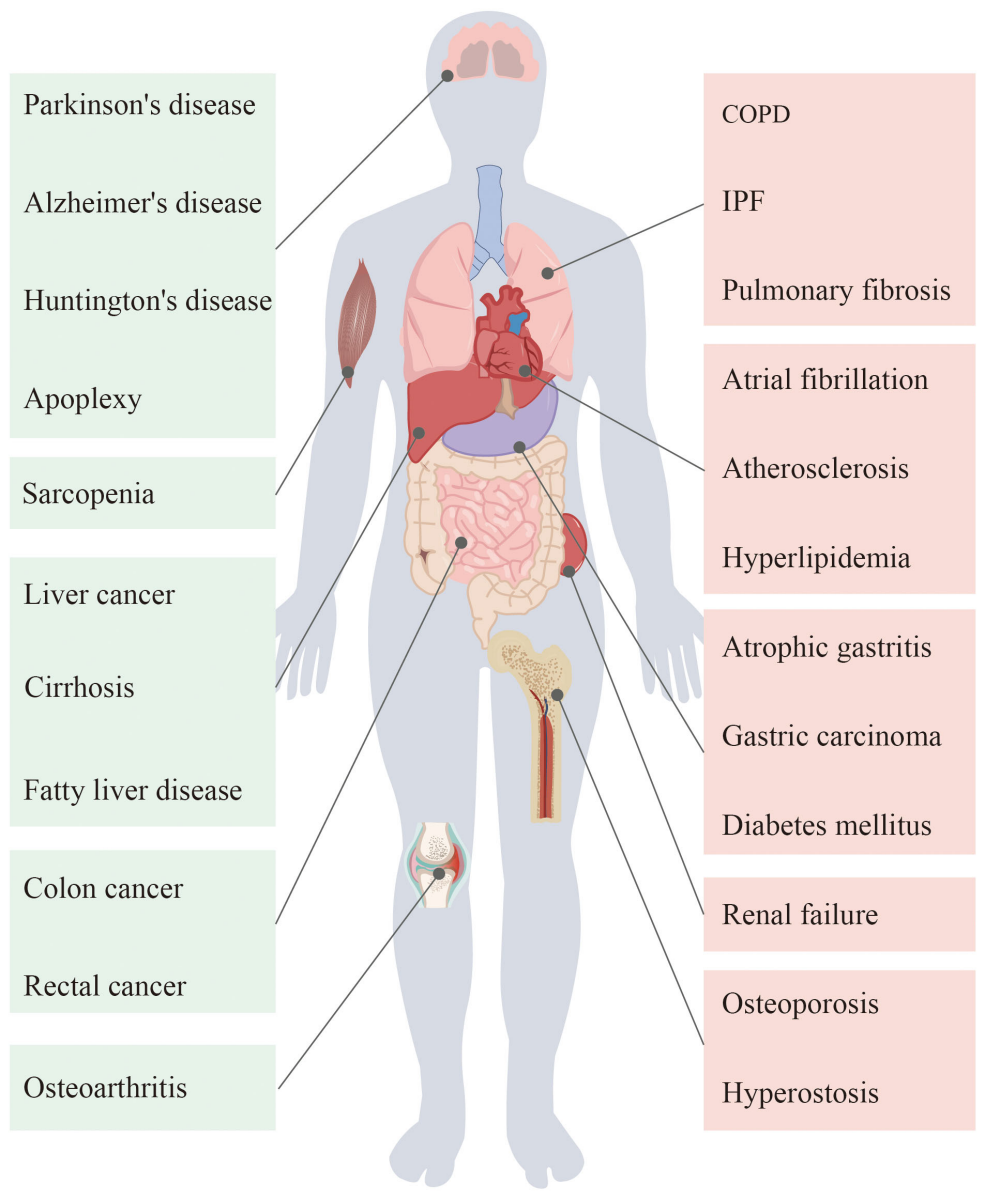
Ageing in the human body organs will appear different degrees of pathological changes, thus causing a variety of diseases.

Ageing occurs as a result of the interaction of countless factors, and it has extremely complex biological behaviors. Cellular ageing, telomere loss, genomic changes, and dysregulation of mitochondrial function drive the progression of ageing and promote the development of related diseases (1). The organs of the human body are altered in various ways by ageing (**Figure 1**). With the development of the ageing population, the study of ageing has become a hot topic of clinical research. Investigations into the molecular mechanisms of ageing have also greatly advanced.

Progressive declines in tissue and organ function are major features of ageing and represent a significant manifestation of musculoskeletal system disease. In people over 65 years of age, chronic musculoskeletal system disorders are the most common conditions leading to impaired physical frailty mobility (2). A large number of studies have shown that ageing is a clear cause of a series of musculoskeletal system diseases, such as osteoarthritis, osteoporosis, and sarcopenia (3). Some studies suggest that the cellular senescence secretory phenotype (SASP) involves a variety of cellular immune factors and enzymes caused by the occurrence of “ageing inflammation”, resulting in changes in the local microenvironment of the body and leading to cartilage and other connective tissue damage, thus causing a wide range of musculoskeletal diseases in elderly people.

Physiological ageing of the musculoskeletal system is characterized by loss of muscle strength and muscle mass, known as sarcopenia, bone loss or osteoporosis, and destruction of cartilage or osteoarthritis (OA) (4–6). The occurrence and development of musculoskeletal system-related diseases have become the most common disabling factors, resulting in a serious burden on social health care. In this paper, we will focus on the role of cellular senescence in musculoskeletal-related diseases and provide a basis for further research.

## 2 Mechanisms of ageing

**Figure 2:**
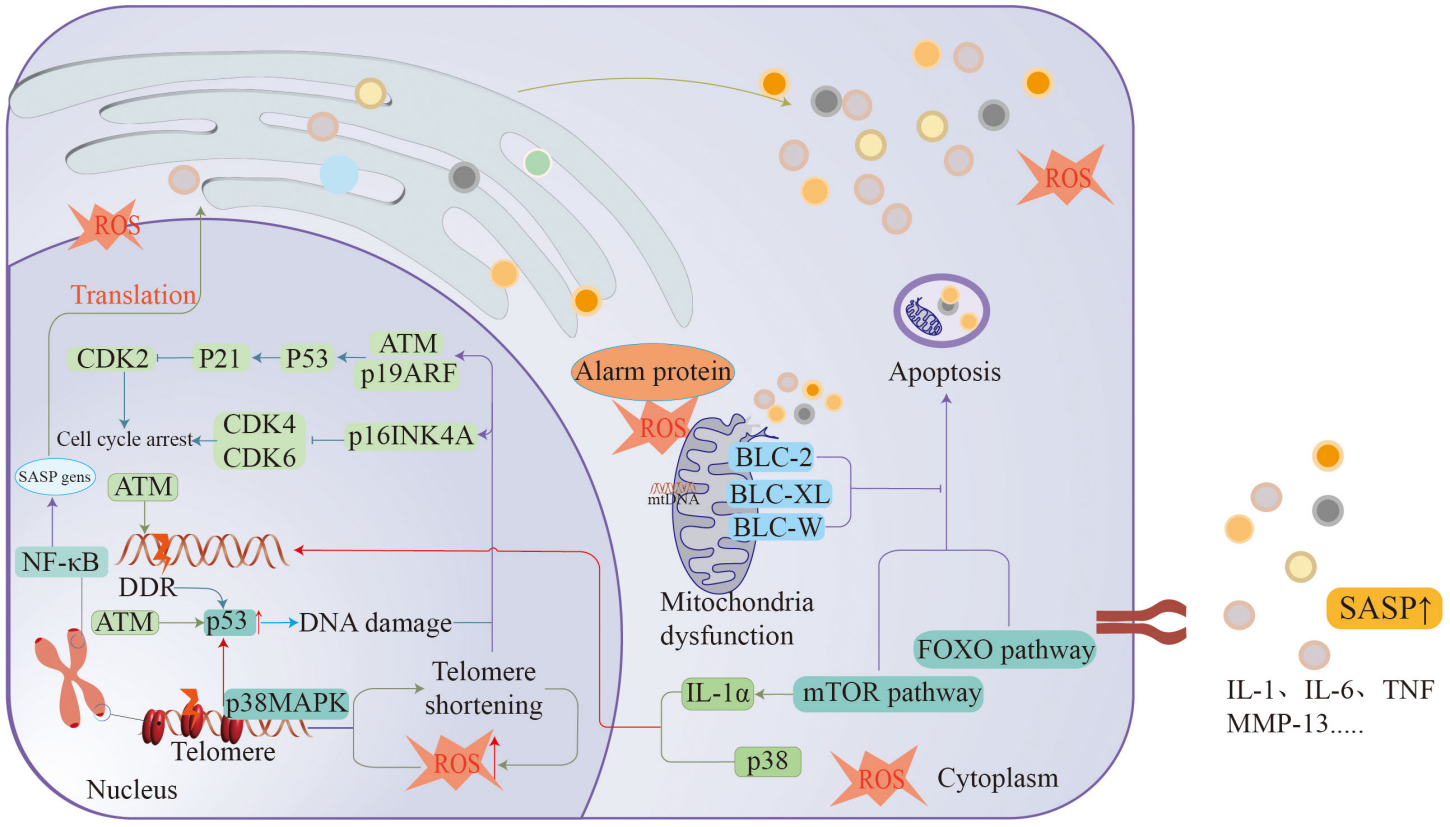
Ageing is associated with many mechanisms, such as DDR, cellular senescence, macrophage dysfunction, telomere loss, etc., all of which cause arrest of the cell division cycle to promote the formation of ageing.

The impact of ageing on the human body is comprehensive and complex, affecting various systems of the human body and leading to functional deterioration and even the occurrence of disease. With the deepening of ageing research, the mechanism of ageing has gradually surfaced. The mechanism of ageing is intricate and closely interrelated, affecting the state of the entire body (**Figure 2**).

### 2.1 Cellular senescence

Cellular senescence was first proposed by Hayflick and Moorhead in 1961 (7). Senescent cells undergo irreversible division arrest and gradually accumulate over time to cause dysfunction in the body. Shortening of telomeres, induction of oncogenes, and impairment of mitochondrial function can lead to the development of cellular senescence (8). Despite the arrest of ageing growth, the effects of cellular senescence on the body are clearly correlated with the altered performance of ageing cells (9). In some cases, cellular senescence plays an active role in the normal physiological activities of the body, and induced recovery of cytokines secreted by senescent cells can be observed during wound recovery. Ageing cells are able to exert anticancer effects by limiting the replication of precancerous cells (10–12). Moreover, the occurrence of cellular senescence also plays an important role in the physiopathological mechanisms of many age-related diseases.

Cellular senescence is a stable state of growth arrest, but it is a dynamic process during which cellular senescence occurs and is induced by many molecular pathways (13, 14). Cellular senescence involves complex mechanisms. DDR, telomere shortening and damage, activation of the INK4/ARF locus, induction of the p53/p21CIP1 pathway, and chromatin reorganization have all been shown to be associated with the development of cellular senescence (14–16). The DNA damage response (DDR) is considered to be a common factor in the development of cellular senescence. When DNA is damaged, DDR factors accumulate at the sites of damage, and cellular senescence is caused by entrapment of the cell division cycle into an arrested state through DDR signaling kinases (ATM, ATR, CHK1 and CHK2) (17–19). Moreover, the activation and expression of the related tumor suppressors p53, p21, and p16 also play important roles in the process of cellular senescence (20–22). As a means of fighting cancer, oncogene expression also induces cellular senescence. Over proliferation triggered by the expression of oncogenes is associated with altered DNA replication status and induces cellular senescence through participation in the DDR pathway (23, 24). Activation of the p53 and p16INK4a/Rb tumor suppressor networks is also a major factor in cell cycle arrest, reducing CDK2 activity through transcription of p53-inducible (CDKi) p21CIP1, allowing cells to exit the cell growth cycle (15, 25, 26). These activated networks intervene in the development of cellular senescence by hindering p53 expression (27, 28). The occurrence of cellular senescence is a mechanism by which the body inhibits the development of cancer. The activation of the INK4/ARF locus is another mechanism by which cells undergo senescence; the INK4/ARF locus encodes three tumor suppressors: ARF, p16INK4a, and p15INK4b. These pathways can hinder the degradation of p53 and affect cellular senescence (29, 30). P16INK4a is also often considered the most important cellular marker of cellular senescence (31, 32). Changes in some organelles can also cause ageing. Impaired ribosome biogenesis is an important factor in cell cycle arrest, excessive rRNA transcription in ageing cells or rRNA processing inhibition in replicative ageing can produce nuclear stress, and the accumulation of the ribosomal 40S subunit protein RPS14 can allow the cell cycle to fall into a state of arrest through the CDK4/rb pathway (33, 34). The RPL22/eL22 protein in ribosomes can then prevent the phosphorylation of RB and promote cell cycle arrest through the inhibition of CDK4-Cyclin D1 (35).

Cellular senescence also exerts biological effects in the local cellular environment or on the entire body, and the sum of various cytokines produced by senescent cells is called the SASP (36). Although the SASP is different during cellular senescence in different tissues, a large number of *in vitro* cellular senescence assays have reported that the SASP is composed mainly of IL-6, IL-8, and MCP1 and contains a variety of proinflammatory factors and various ECM remodeling-related enzymes (37–39). In recent years, proteomic analysis of the SASP has revealed a number of different SASP effectors in the human body that can act as soluble molecules or be released into the extracellular space via exosomes; moreover, many age-related diseases are associated with enrichment of the SASP in plasma (40). The SASP is regulated at multiple levels, and sustained signal transduction by the DDR is key to SASP-related inflammatory molecules, with ATM, NBS1, and CHK2 being the main regulators (41, 42). The SASP is regulated by many pathways and depends on various secretion pathways to regulate cell and tissue ageing. The characteristics of the SASP are also very different under different signaling pathways. The DDR and cGAS/STING pathways are related to SASP regulation. The SASP affects the development of cellular senescence through the regulation of various cascade reactions (43, 44).

### 2.2 Telomere attrition

Telomere shortening is a classic mechanism of ageing. In telomere attrition, as cells replicate and proliferate, telomeres shorten as the number of divisions increases, leading to the development of senescence (45, 46). Telomere shortening and dysfunction have been shown to be significantly associated with age-related diseases in many studies. In many tissues and organs with high proliferative activity, the continuous division of cells leads to gradual shortening of telomeres and gradually leads to abnormal changes in DNA. These changes can lead to a series of physiological changes, such as ageing, apoptosis, and blocked cell differentiation, to induce disease. However, in tissues and organs with a low proliferative tendency, telomeres can also be affected by factors such as ROS, leading to telomere damage. Oxidative stress can affect skeletal muscle, cardiac muscle and other cells to accelerate ageing and cause related diseases (47, 48). Telomere shortening is also strongly associated with ageing-related inflammation, and the development of ageing and chronic inflammation can lead to the development of diseases such as osteoarthritis, atherosclerosis, and type II diabetes. Inflammatory ageing caused by telomere loss and dysfunction is mainly driven by the secretion of high ROS, and high levels of ROS and an inflammatory response will continue to aggravate the loss and imbalance of telomeres, resulting in a malignant cycle (49, 50). Therefore, inhibiting the increase in ROS by maintaining mitochondrial activity is the main treatment for ageing-related diseases (49).

### 2.3 Inflammation

Inflammation often runs through aging as an important clue. Inflammation can occur widely in aging tissue cells and induce immune responses, leading to the development of diseases. The development of chronic inflammation is also considered one of the hallmarks of the development of aging (51, 52).

Chronic inflammation and aging are mutually influential processes. senescence-associated secretory phenotype (SASP) induces senescence in normal cells through the development of chronic inflammation. At the same time, chronic inflammation promotes aging of related immune cells resulting in the inability of people to timely remove aging cells and related inflammatory factors. Thus forming a malignant cycle causes further development of aging. At the cellular level, mitochondrial dysfunction and DNA damage and oxidative stress can cause chronic inflammation. These senescent cells secrete SASP that includes most of the inflammatory mediators such as IL-1β and IL-6 as key factors that promote the development of inflammation (53, 54). Aging-related chronic inflammatory responses are mainly endogenous inflammatory responses formed by endogenous cellular molecular junk stimulation (55, 56). Oxidative stress can induce systemic inflammatory response through signaling pathway by inducing the damage of intracellular biological factors. It also promotes oxidative stress through increased secretion of inflammatory mediators. Damage to the genome by ROS, prompting telomere shortening DNA fragmentation has been identified as a key factor in the development of cellular senescence These damaged DNA fragments leak into the cytosol directly causing inflammation (57).

### 2.4 Disruption of macroautophagy

Autophagy is a metabolic process in which various cellular components are degraded and recovered, and the stable operation of cells is maintained by the phagocytosis and decomposition of abnormal macromolecules and organelles (49). A large body of evidence suggests that a decline in autophagy is associated with the development of ageing and related diseases. With increasing age, autophagy naturally decreases. Simultaneously, stalled autophagic vesicles can accumulate with age, resulting in a systemic decline in autophagic activity (58, 59). The upstream regulatory function of autophagy disorder is mainly realized by the insulin/IGF-1 signaling pathway, which affects the level of autophagy activity through the combination of FOXO and the sensing regulation of mTOR trophic factors and growth factors. The activity of the transcription factors TFEB and FOXO is also affected when the insulin/IGF-1 signaling pathway is dysregulated, and these changes reduce autophagy and lysosome-related expression (60, 61). These regulatory pathways are vulnerable to age factors. Changes in mTOR activity can have a significant impact on autophagic flux, and increased mTOR conduction can be significantly observed in the muscle tissues of aged mice and humans (62). Dysregulation of FOXO activity induces musculoskeletal-related diseases, and autophagic gene expression is significantly lower in the cartilage of elderly osteoarthritis patients, demonstrating that reduced autophagic activity in cartilage tissue leads to the development of cartilage degeneration (63, 64).

### 2.5 Mitochondrial dysfunction

The mitochondria play a role as power providers in the normal physiological activity of cells, but mitochondria also play roles in the development of inflammation and cell death (65). Mutations in mtDNA can accumulate within mitochondria with age, resulting in the dysregulation of mitochondrial oxidative phosphorylation, increasing ROS production and thereby causing cellular senescence (66). According to related experiments, the accumulation of mtDNA mutations and deletions results in significant ageing phenotypes, including sarcopenia, osteoporosis, shortened lifespan, and decreased fertility (67, 68). Instability of mtDNA within mitochondria can also contribute to the development of ageing. mtDNA tends to replicate more frequently during physiological activities and is present in an oxidative environment with mitochondria. These conditions place mtDNA at a greater risk of mutation and deletion (69). Increased mtDNA mutations can be observed in various ageing biological tissue cell systems. Recent studies have shown that mtDNA is also exported only extracellularly and is detected in cerebrospinal fluid as a biological signal interconnected between mitochondria in distal tissues. Decreased secretion of melatonin in elderly individuals prompts mtDNA release from mitochondria and activates the cGAS/STING/IRF3 signaling pathway to produce inflammatory cytokines involved in the development of cellular senescence (70). Increased mtDNA mutations can be observed in various aging biological tissue cells. Recent studies have found that mtDNA also exports only extracellularly and is detected in cerebrospinal fluid as a biological signal interconnected between mitochondria in distal tissues. Decreased secretion of melatonin in the elderly prompts mtDNA release from mitochondria and activates the cGAS/STING/IRF3 signaling pathway to produce inflammatory cytokines involved in the development of cellular senescence (71–73). Mitochondrial dysfunction also leads to the development of chronic inflammation. Some immune factors are released in response to cell damage during the development of immune responses, and the related factors are called alarm proteins (74, 75). The immune factors include mitochondria-associated molecules, such as mtDNA, ATP, succinate, and ROS, and these mitochondria-derived molecules induce inflammation through different pathways (76). Among them, mtDNA has already been confirmed to be the strongest immune activator. Several PRRs bind mtDNA to trigger innate immune pathways to induce inflammation; for example, TLR9 binds mtDNA to stimulate NF-κB signaling pathways to stimulate inflammation development (77, 78). During organ transplantation, older donor organs can lead to aging of the transplanted person. After aging organ transplantation, the expression of SASP in recipients will be significantly increased to induce the aging of young cell cells, and even the aging cell population. Accumulation of mtDNA also greatly reduces the survival rate of transplanted organs (79, 80). In the experiment of *Jasper Iske et al.*, senolytics therapy was effective in improving the survival probability of transplanted organs in recipient mice after treatment of aging donor mice. It also reduces the level of inflammation in recipient mice after transplantation (81). Mitochondrial dysfunction leading to chronic inflammation is a causative factor in many diseases, and targeted mitochondrial therapy via pharmacological intervention can effectively treat age-related diseases (66).

### 2.6 Protein homeostasis

Most of the physiological functions of humans are inseparable from proteins, and the damage and inactivation of normal proteins in the human body can lead to the occurrence of a series of related diseases (82). Many neurological diseases are mainly associated with protein damage. To prevent protein denaturation, cells prevent protein misfolding by producing chaperone proteins. When the structural homeostasis of proteins changes, autophagy and the unfolded protein response (UPR) together constitute a clearance system for abnormal proteins (83). The transcription factors ATF3 and ATF4, which regulate UPR activation, therefore play important roles in the processing of abnormal proteins. Abnormal expression levels of ATF3 and ATF4 lead to failure of the ubiquitination of abnormal proteins that accumulate protease targets to accelerate ageing and the development of related diseases; moreover, it has been shown that reducing the concentration of ubiquitinated targets can prolong lifespan (84–86). A newly discovered ubiquitin-like molecule, SUMO, can improve stability by modifying proteins. Silencing of SUMO-encoding genes has been found to lead to a reduced lifespan in *C. elegans* (87, 88). In addition, insulin-like growth factor-1 (IGF-1) inhibits the degradation of abnormal proteins by regulating the autophagy–lysosome system and inhibiting lysosomal protein degradation, such as that of the proteasome. IGF-1, a well-established ageing factor, can significantly inhibit ageing by inhibiting the IGF-1 signaling pathway (89). However, in the musculoskeletal system, certain levels of IGF-1 can promote muscle cell synthesis of proteins through the PI3K/Akt/mTOR and PI3K/Akt/GSK3β pathways and lead to a decrease in muscle strength when IGF-1 levels are too low (90, 91).

### 2.7 Genome instability

Genomes are frequently affected by various exogenous and endogenous factors. These effects often cause damage to a range of genetic materials. In response to these injuries, organisms have evolved a series of damage repair mechanisms. Damage to DNA activates the DNA damage response, preventing the transmission of damaged genetic information to offspring cells through the replication of damaged DNA in tissues. However, with age, these repair systems gradually fail, and damaged genes continue to accumulate, leading to ageing of the body (92, 93). *Tabula et al.* confirmed a positive correlation between the generation of genetic mutations and the development of ageing through the analysis of a large number of mouse cells of all ages (94). Improving the stability of the genome can effectively delay the occurrence of ageing. In an experiment, overexpression of SIRT6 in mice effectively improved the stability of the genome and prolonged the lifespan of the mice (95).

### 2.8 Epigenetic changes

Epigenetic changes such as DNA methylation, abnormal histone modification, and abnormal chromatin remodeling promote ageing. These changes can lead to a range of ageing-related diseases, including various cancers, metabolic diseases, neurological diseases, and musculoskeletal system diseases. DNA methylation in the human body mainly refers to the regulation of gene expression by the binding of DNA to proteins that inhibit transcription (96). Methylation, a stable DNA modification, can be used as an epigenetic marker (97). According to related studies, there are fewer DNA methylation modifications in senescent cells than in permanent and proliferating cells, and DNA methylation gradually decreases during cellular senescence. Steve Horvath proposed the concept of the epigenetic clock through methylation and age, and Levine proposed the theory of DNAm PhenoAge. These studies demonstrate a relationship between the epigenetic clock and ageing and indicate that ageing-related alterations can be deduced by DNA methylation levels (98, 99). Histone modifications are additional major forms of epigenetic alterations. Loss of histones and modification of histones can also be clearly observed when ageing occurs, with the greatest changes being methylation and acetylations (100). Histone modifications can be involved in the regulation of transcriptional function. In related studies, patients with Hutchinson-Gilford syndrome and Werner syndrome exhibited significant chromatin abnormalities and significant decreases in H3K9me3 or SUV39H (H3K9me3 histone methyltransferase) levels (101). Reducing the activity of histone acetyltransferases prolongs the lifespan of progeria mice (102).

### 2.9 Deregulated nutrient sensing

Nutrient sensing is the ability of human cells to recognize nutrients such as glucose and is regulated mainly by the insulin/IGF-1 signaling, mTOR, and AMPK pathways. Nutrient-sensing pathways can regulate the absorption and distribution of nutrients through the state of the cell (103). Various pathway regulatory networks interact with each other to form feedback loops to regulate metabolic processes in the body, such as protein and nucleotide synthesis. These regulatory networks mainly promote anabolic progression at young ages and ageing at old ages (104). The microbiota in the human body is also involved in nutrient sensing to some extent, and the gut–brain axis (GBA) demonstrates that there is communication between the gut microbiota and the brain (105, 106). The intestinal microbiota plays important roles in metabolism and the immune response, and ageing-related microbiota can promote the development of systemic inflammation, leading to the occurrence of ageing-related diseases. During ageing, glial cells undergo a series of disorders leading to neurodegenerative diseases in the brain. In experiments, microglial function was effectively improved by resupplementing mice with relevant gut microbiota metabolites (56, 107).

### 2.10 Stem cell exhaustion

Stem cells have the potential to regenerate and differentiate and are associated with cell turnover and the repair of damaged tissues *in vivo*. Stem cells, like ordinary cells, are ageing cells. Autophagy is an important mechanism for maintaining stem cell activity and differentiation ability. Autophagy activity in MSCs can effectively improve the ageing of stem cells. However, with ageing, decreased autophagic activity naturally causes the differentiation and proliferation of MSCs (108, 109). Ageing triggers a series of processes of asymmetric cell division and differentiation, resulting in stem cell depletion or abnormal proliferation and differentiation. According to related mouse experiments, ageing can significantly affect the division and differentiation of T cells, resulting in premature ageing and impaired T-cell function (110).

### 2.11 Changes in intercellular communication

The normal physiological activities of the human body are inseparable from the transmission of information, and the body’s complex and large communication network links various organs, tissues and even cells to form a whole. Intercellular communication occurs mainly through the exocytosis of signaling factors and other means to affect adjacent cells (111). The SASP causes inflammation and senescence in normal cells through its effects on adjacent cellular functions. Senescent cells in tissues mainly communicate between cells through juxtacrine signaling, cell fusion, cytoplasmic bridges, and extracellular vesicles, causing the “spread” of ageing in the local or even systemic circulation (112, 113).

## 3 Role of ageing in musculoskeletal system diseases

**Figure 3:**
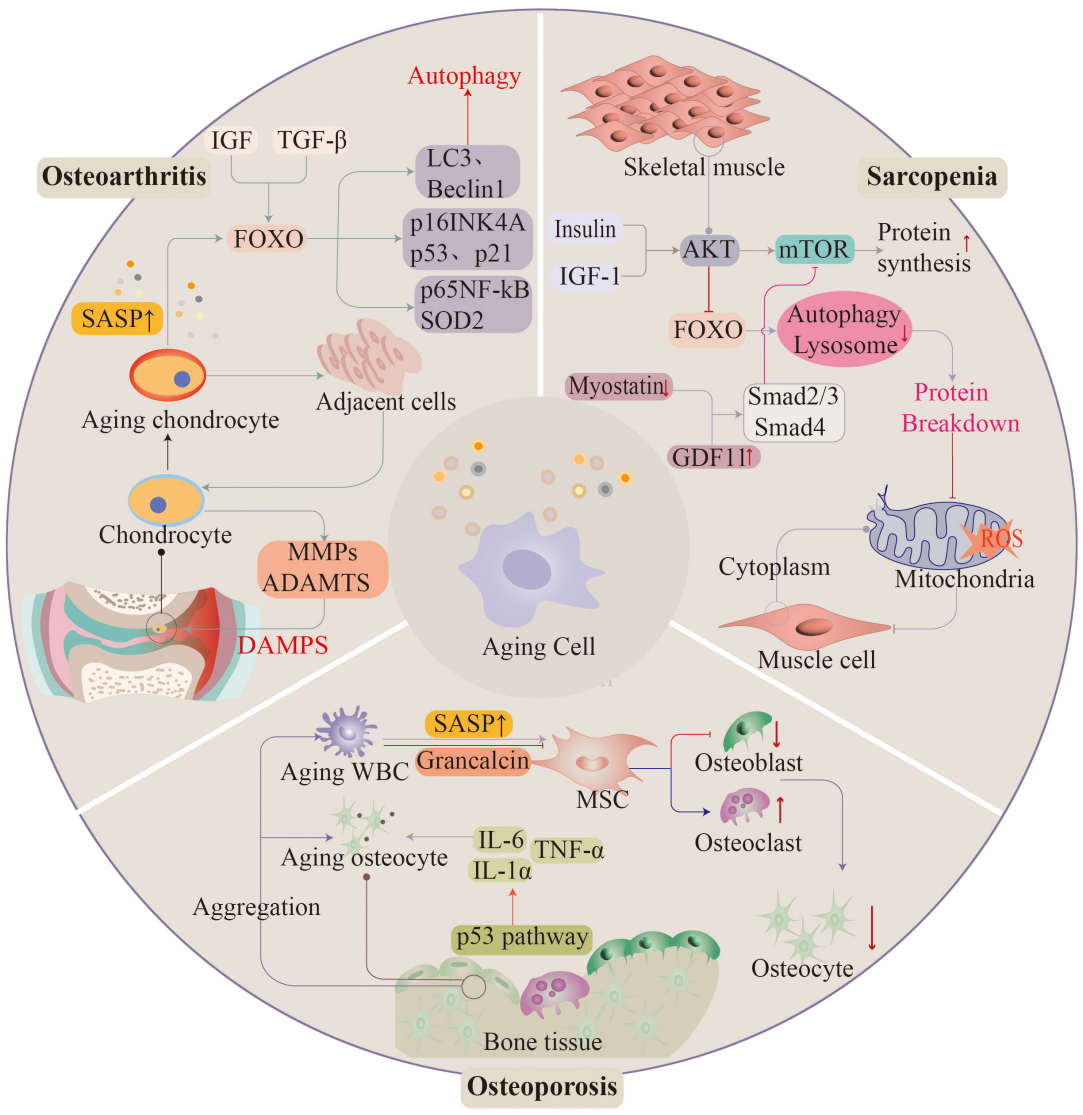
As ageing progresses, the musculoskeletal system often appears in significant functional deterioration. Osteoarthritis, osteoporosis, and sarcopenia are associated with ageing. Ageing of histiocytes promotes the development of these diseases.

Diseases of the musculoskeletal system occur widely in the elderly population, with approximately 1.71 billion people worldwide suffering from musculoskeletal disorders. Diseases of the musculoskeletal system have a great impact on the quality of life of the population, severely limiting the patient’s mobility and the ability of the labor force to engage substantially in social activities. As the population ages, the number of diseases will continue to increase in the future (114) (**Figure 3**).

## 4 Ageing and osteoarthritis

Osteoarthritis (OA) is a chronic degenerative disease that occurs within joints and is mainly characterized by cartilage degeneration and osteophyte formation in patients, often accompanied by joint pain, difficulty in movement and other symptoms, mostly in elderly patients (5, 83). As the most common joint disease, osteoarthritis causes not only physical pain but also mental pain. There are many risk factors for the development of osteoarthritis; obesity, a history of joint injury, sex, and genetics are all causative factors for its development, though the most important risk factor is age (115).

The development of osteoarthritis is closely related to the development of cellular senescence. Cellular senescence in articular cartilage is not only age-related, but mechanical damage or mechanical stress stimulation at the joint can also lead to the occurrence of chondrocyte senescence. The most common pathogenesis of osteoarthritis suggests that the occurrence of osteoarthritis is associated with the occurrence of subchondral sclerosis (116, 117). With respect to cartilage destruction, progressive loss of articular cartilage is a hallmark injury in OA. Cartilage damage is almost unavoidable with age, which in turn progresses to the bones and joints. Although chondrocytes have always been recognized as nonregenerable, according to some studies at the cellular level, chondrocytes can regenerate after articular cartilage injury, and the cartilage matrix proliferates in clusters during the early stages of OA (118). At the cellular level, cartilage organizers include a group of cells with stem cell functions, namely, CSPCs, which have a certain positive effect on cartilage injury. Cellular ageing has a certain positive effect on cartilage mechanisms. In addition to the ageing damage of chondrocytes themselves, the ageing of chondrocytes in OA causes changes in the secretory phenotype of other cells in the joint, that is, the effect of “SASP” paracrine signaling (119). Transplantation of senescent fibroblasts into the articular cavity in a mouse model resulted in cartilage damage, leading to changes in the cellular environment in the synovial membrane of the articular cavity and inducing OA (120). Elevated levels of IL-1α, IL-6, MCP-1 and other factors in synovial fluid can also be caused by cells other than chondrocytes (121). For example, premature senescence can be induced in normal human fibroblasts by IL-6–STAT3 signaling (122). Obviously, osteoarthritis development is not influenced by only one cytokine. However, there is relevant evidence that the secretion of ageing phenotypes associated with chondrocyte injury (e.g., IL-6, IL-1α) has an impact on the development of OA. However, because these cytokines are also present in osteoarthritic chondrocytes, the types of cells that cause the abnormal expression of these mediators has not been determined (123). Gene expression patterns can also change in articular chondrocytes as a result of increased SASP in osteoarthritic tissue cells with ageing. Growth factors are released directly or indirectly by chondrocytes during osteoarthritis, and high levels of synthesis and release promote downstream BMP-2 expression and stimulate extracellular matrix turnover (124). The ageing-induced release of cytokines from chondrocytes or other cells in the joint cavity induces the upregulation of MMP expression, leading to the degradation of ECM cartilage, thereby driving the development of osteoarthritis. MMP-13 can cut type II collagen, and in cartilage injury, cartilage breakdown products stimulate the secretion of MMP13 by related cells to further loss of cartilage and accelerate the development of OA (125). Through the transformation of related cellular ageing phenotypes, the chondrocyte ageing pathway, the upregulation of β-galactosidase production, the increase in p16 expression, the irreversible growth arrest of related cells, and the increase in extracellular vesicle secretion are all involved in the transformation of cell phenotypes that are strongly related to the development of osteoarthritis (126). Currently, cartilage damage caused by cellular senescence remains a major topic of research. Osteoarthritis is often treated clinically by repairing articular cartilage or injecting matrix into the joint cavity to relieve the progression of osteoarthritis, such as with intra-articular stem cell injection. The important role of cell senescence injury-related factors in the development of osteoarthritis has been confirmed. Studying the mechanism underlying the relationship between cellular senescence and the occurrence of osteoarthritis can provide more options for the treatment of cartilage injury in osteoarthritis or for delaying the progression of osteoarthritis and improving the quality of life of affected patients. Analyzing the process by which arthritic chondrocytes accumulate damage from the perspective of cellular ageing under the mechanism of SASP and identifying the targets of cytokine signaling pathways in the ageing process can reveal more effective methods for the treatment of osteoarthritis.

As SASP research progresses, a major goal of ageing research is to better understand common mechanisms that can delay loss of function in multiple systems, which will lead to improved “healthy longevity” rather than simply increasing longevity. Heterochronous symbiosis experiments combining the circulatory systems of juvenile and aged animals have revealed systemic factors that alter the ageing phenotype, including growth and differentiation factor 11 (GDF11), oxytocin, and IL-15, which may reduce ageing in multiple organ systems. An important aim of future studies will be to determine whether these ageing-related factors affect OA development.

## 5 Ageing and osteoporosis

OP is a disease characterized by bone loss and microstructural changes, and its occurrence is strongly correlated with age. With the progress of research, the occurrence of OP is also closely related to the occurrence of ageing. When ageing occurs, the release of large amounts of SASP products and other effects will lead to disruption of the balance of bone homeostasis, and changes in the local microenvironment or systemic factors will lead to the occurrence of osteoporosis (127, 128). Treatment of ageing cells has been shown to significantly improve bone loss in mice (129).

Osteocytes account for 90% of all bone-related cells and play important roles in the normal physiological structure and activity of bone. An increasing number of related studies have shown that osteocyte ageing is significantly associated with bone metabolism disorders (130). Senile osteoporosis is mainly characterized by the loss of cortical bone and trabeculae, which are mainly reflected in the loss of cortical bone during ageing (131). Osteocyte and osteoblast dysfunctions are considered to be the main causes of bone loss in elderly patients with OP (127). Ageing occurs in osteocytes for a variety of reasons: in addition to replicative ageing associated with telomere shortening during the cell cycle and body ageing, cytotoxic stress is induced; oncogenes are stimulated; free radical accumulation occurs; reactive oxygen species are generated; DNA damage, telomere damage, protein arrest, and mitochondrial dysfunction occur; and prosurvival pathways are activated (132–134). Changes in the expression levels of some genes, such as WNT10B, RUNX2, RANKL, Osterix, osteocalcin, osteoprotegerin(OPG), and Sclerosteosis(SOST), have revealed these effects, which sometimes differed in male patients with idiopathic osteoporosis, and their expression levels tended to decrease (135). Studies using RNA sequencing technology have assessed genes that change with age-related osteoporosis and revealed approximately 700 DEGs as well as 12 cellular pathways related to skeletal ageing (136).

With increasing age, ageing osteocytes and bone marrow cells upregulate the expression of multiple SASP factors in the bone microenvironment of aged mice, and the expression of the key ageing marker p16INK4a doubles with the ageing of bone-derived B cells and bone marrow T cells, osteoprogenitor cells, osteoblasts, and osteocytes (137, 138). The upregulation of several SASP-related mRNAs, such as p16INK4a and p21, was also evident from the comparison of the expression of related factors in elderly women, and the expression levels of SASP effectors were significantly greater in elderly women than in young women (139). The bone microenvironment of aged mice shows obvious ageing characteristics, and the occurrence of OP is strongly associated with the balance of osteoblasts and osteoclasts. Osteoblasts and leptin receptor-positive cells, which are the main source of osteoblasts, also senesce with increasing age in mice (140–142). This process leads to changes in osteoblast differentiation to adipocyte differentiation, which triggers senile osteoporosis (143, 144). Some studies have shown that p6 and p21 mRNA levels are significantly increased in osteocytes of aged mice (145). When ageing occurs, the levels of two autophagy marker genes (Atg7 and Map1lc3a) in the osteocytes of aged mice decrease, which indicates a reduction in mitophagy in mice, and the damaged mitochondria cannot be removed in time, leading to the generation of more reactive oxygen species (ROS) and subsequently contributing to ageing (139, 146). Although senescent osteocytes account for only a very small proportion of the total number of osteocytes, SASP release by these senescent osteocytes induces an inflammatory microenvironment in bone, resulting in disturbances in bone metabolism (145). Some specific SASP factors, such as TNF-α, IL-1 and IL-6, promote the ageing of nearby healthy cells and lead to bone resorption and the inhibition of bone formation; the resulting bone loss then induces OP. Treatment of mouse bone marrow cells with senescent cell culture medium has revealed that osteoclast differentiation is significantly increased in mice (129). Increased SASP-related senescence can significantly affect normal cells. There are also factors related to the SASP that can regulate gene expression in osteocytes and inhibit osteogenic differentiation and mineralization. Previous studies have shown that blocking the SASP and the secretion of MLO-Y4 cells can relieve the inhibition of bone mesenchymal stem cell (BMSC) differentiation and improve the occurrence of OP (147, 148). In clinical practice, cortical bone ageing loss is more severe than trabecular bone loss in patients with primary OP, suggesting that bone loss in ageing osteocytes may involve different mechanisms at different bone sites (149, 150).

Another important player in bone homeostasis is the immune system. According to *Farr et al.*, immune cells also play an important role in the development of senile OP (137, 151). Neutrophils and macrophages undergo senescence in aged mice, and senescent macrophages also release SASP factors to promote M1 polarization and ageing in other macrophages (137, 139, 151). Large amounts of grancalcin are accumulated and secreted from the bone marrow, which bind and inhibit the signal transduction of the b2 receptor of MSCs, reducing osteogenesis (152). The ageing of immune cells is a potential factor in the development of senile OP. In alveolar bone, repeated stimulation of LSP triggers the activation of p53 and increases the expression levels of IL-1α, IL-6 and TNF-α, which in turn causes DNA damage and induces cellular senescence in alveolar bone, resulting in the secretion of local SASP to form OP (41, 153, 154).

Thus, the ageing of a variety of cells in the bone microenvironment can cause bone loss, and although the ageing of myeloid cells and osteocytes are predominant, the ageing of other cells still has a certain impact on the occurrence of OP (139).

Estrogen has a considerable effect on the metabolism of osteocytes and osteoblasts and can alleviate the ageing of osteocytes and osteoclasts to a certain extent (155). Therefore, changes in estrogen levels in the human body can have a certain degree of impact on the metabolism of osteocytes and osteoclasts (156). After menopause, a large decrease in estrogen levels leads to accelerated proliferation and metabolism of osteoclasts and stimulates bone resorption on trabecular bone surfaces. This process stimulates the activity of osteoclasts, causing the upregulation of SOST. The upregulation of SOST inhibits WNT signaling, increases osteoblast number, and decreases osteoblast activity (127). This causes an imbalance in bone metabolism to form OP. With the ageing of the body, bone cell ageing can lead to OP. Estrogen plays an important role in bone cell ageing. In OVX mice, the proportions of p16 and β-galactoside 127 in bone cells are high, and they produce effectors such as MMP-3, MMP-13, IL-6, IL-8, IL-1α and IL-1β. Other SASP component-related experiments have also demonstrated that exogenous estrogen supplementation is quite effective for bone loss in OVX mice; related studies have shown that exogenous estrogen supplementation can effectively alleviate osteocyte senescence in OVX mice when exogenous estrogen supplementation is available. By hindering p16 and other related signaling pathways, bone loss in OVX mice is substantially attenuated or prevented (157, 158). However, at present, the specific mechanism by which estrogen delays osteocyte senescence is not clear, and relevant studies have shown that this effect may be related to the inhibition of Usp10 by estrogen (159). A related study from 2019 showed that a decrease in estrogen levels did not have an adequate effect on bone cell SASP concentrations (160). Bone loss caused by estrogen deficiency may not be caused by decreased estrogen levels, which directly affect the cellular ageing process. It is more likely that estrogen deficiency leads to changes in cellular microenvironment-related factors that affect the ageing process of osteocytes, such as promoting their ageing by inducing oxidative stress.

Age-related bone loss can be prevented by eliminating senescent cells or blocking cellular senescence-associated pathways (129). Aged mice clearly showed improvements in skeletal microarchitecture and strength after 4 months of treatment with anti-ageing drugs (eliminating senescent cells) or 2 months of treatment with JAK inhibitors (blocking the proinflammatory secretome of senescent cells) (161, 162). However, IL-1a and Tnfsf11 mRNA could significantly decrease osteoclast numbers and increase osteoblast numbers via the effective clearance of senescent osteocytes (163, 164).

## 6 Ageing and sarcopenia

Sarcopenia is a disease characterized by the loss of muscle strength and mass (165). The musculoskeletal system allows movement and protects vital organs; over time, its erosion leads to an increased risk of fractures and physical weakness, which are hallmarks of ageing (166).

Cellular senescence, to some extent, can be regarded as inflammation at the molecular level in the human body, causing a range of age-related diseases as a major consequence of ageing due to DNA damage (167). In ageing mice, symptoms of sarcopenia are often observed, and elevated expression levels of SASP factors such as Igfbp2, MMP13, and Pai1 are clearly detected in their bones (168). Ageing can have a significant effect on the number of skeletal muscle cells, and SASP-related factors were significantly increased in the skeletal muscle cells of older rhesus monkeys (169, 170). The artificial induction of ectopic p16Ink4a expression in aged mice inhibited the proliferation of damaged skeletal muscle satellite cells. However, inhibiting the expression of p16Ink4a can significantly alleviate sarcopenia in ageing mice (168, 171). The number of skeletal muscle satellite cells decreases with increasing age. Moreover, skeletal muscle satellite cells in aged mice develop fibrosis by activating the Wnt signaling pathway, which in turn leads to fibrosis of skeletal muscle tissue (172). Increased cellular senescence and inflammatory responses can also lead to muscle loss by damaging stem cells. Inflammatory responses occurring throughout the body are also associated with decreased function of associated progenitor cells (161, 173). OPA1 regulates the metabolic level of muscle stem cells through FGF21, and muscle loss occurs when OPA1 is reduced. Cultivation of senescent mesenchymal progenitor cells with myoblasts in the experiment resulted in accelerated muscle loss. The expression of proinflammatory factors in the SASP is an important factor in sarcopenia, and the overexpression of IL-6 can significantly lead to muscle atrophy in mice (174, 175).

In recent years, researchers have determined that skeletal muscle is not only the motor unit of the human body but also has a certain immunoregulatory function, Skeletal muscle cells can regulate human immune function by releasing signals such as related cytokines (176). Muscle tissue can release more than 300 cytokines, one of which was previously identified as IL-6 (177, 178). IL-6 also acts as a member of the SASP, causing sarcopenia through proinflammatory effects (179, 180). IL-6 induces muscle atrophy by reducing muscle synthesis and may directly mediate muscle catabolism. IL-6 can also induce muscle atrophy by stimulating abnormal STAT3–IL-6 signaling (181, 182). When IL-6 is administered to mice with the corresponding antibody, muscle conditions can be effectively improved (183). Another apparent correlate is IL-15, a cytokine that is expressed in a variety of tissues, including muscle, and is a part of SASP (178). When synergized with IGF-1, IL-15 induces the synthesis of myosin heavy chain and promotes muscle recovery by inhibiting the differentiation of FAPs into adipocytes (184). The overexpression of FoxO also increases muscle protein breakdown and promotes ageing- and obesity-related muscle degeneration. IL-5 mRNA levels were substantially increased after recovery in subjects trained for high-intensity muscle resistance (185, 186). As an energy level sensor within skeletal muscle cells, AMPK is important for IL-15 production following exercise (187, 188). With increasing age, AMPK activity decreases *in vivo*, causing muscle loss in IL-15 signaling pathways (189).

## 7 Targeted ageing therapy for musculoskeletal system diseases

Ageing is inevitable. The incidence of various diseases will also increase with age. The main research ideas currently aim at treating and preventing some diseases by inhibiting the occurrence of cellular senescence. In many age-related diseases, curbing the growth of senescent cells is an effective treatment (190).

Current major ageing inhibition strategies are divided into senolytics and senomorphics. Senolytics are a strategy to promote apoptosis in senescent cells by intervening in various signaling regulatory pathways within senescent cells via the use of related drugs. Normally, senescent cells upregulate the expression of factors that negatively regulate apoptosis (e.g., BCL-2, BCL-W, BCL-X), thereby maintaining a stable state of senescence arrest (13, 191, 192). Senomorphics inhibit the damage of senescent cells to other normal tissue cells mainly by inhibiting the expression of SASP factors in senescent cells. The expression of SASP components secreted by senescent cells is reduced by related drugs, such as rapamycin (mTOR), which inhibits the occurrence of ageing (193–195). The characteristic of Senomorphics is that it can reduce SASP secretion while ensuring cell survival.

Senolytic therapy often has high requirements for the timing of administration during treatment. Inhibition of BCL-2 and BCL-Xl by Navitoclax at the initial stage of inflammation effectively removed senescent p16 stem cells, promoting histiocyte regeneration. The combined use of quercetin and dasatinib eliminated senescent cells in bone tissue and cartilage by stimulating senescence-associated tyrosine kinase targets (116, 196, 197). In addition, intra-articular injection of UBX0101 (an anti-ageing agent) for the treatment of osteoarthritis resulted in the local clearance of ageing cells to alleviate the progression of arthritis by blocking p53/MDM2-induced apoptosis of ageing cells (198). In a recent mouse study, there was a significant decrease in the number of hydroxylated proteins in synovial fluid and cartilage of articular cavities treated with UBX0101, which could reduce oxidative damage to related proteins. The progression of osteoarthritis was greatly slowed in these mice (199). *Laccarin* has shown benefits in the treatment of musculoskeletal disorders such as osteoporosis and osteoarthritis in relevant animal and clinical drug experiments. *Laccarin* can hinder the development of osteoporosis through osteoblast differentiation and inhibition of bone resorption (200, 201).

In the SASP, MMP13 has been identified as the most important factor contributing to the development of osteoarthritis. Inhibition of MMP13 by CL82198 effectively reduced the degradation of type II collagen and reduced the number of osteoarthritis-related lesions (202, 203). AP20187 is a cell-permeable molecule used to dimerize the FK506-binding protein (FKBP) fusion protein, initiate biological signaling cascades and gene expression or disrupt protein-protein interactions, and treatment with AP20187 can reduce the expression of p16Ink4a, thereby improving bone loss (204, 205). The number of osteoblasts and the rate of bone formation increased in mice treated with AP20187, and the amount of adipose tissue in the bone marrow decreased (129).

## 8 Discussion

Aging is a natural process that affects all systems and organs of the human body. It is an inevitable topic that requires careful consideration and understanding. Aging mechanisms are complex, and different factors interact and promote each other. Continued research on aging is important to prevent aging and its related diseases and improve the quality of life of the elderly.

This review summarizes the main mechanisms of aging and their effects in the musculoskeletal system. Musculoskeletal system diseases have a particularly wide impact on the quality of life, and severe cases can lead to loss of mobility causing serious harm to the psychophysiological health of patients. With increasing age, the number of patients involved is expected to continue to increase, placing a serious burden on the public healthcare system. Musculoskeletal system disorders have a large impact on the quality of prognosis by intervening early. In particular, OA, due to the particularity of cartilage tissue, the injured cartilage can hardly be recovered. Therefore, timely relevant treatment in the early stage of OA is the most suitable therapy.

Many current studies are limited to animal and cellular assays. In these studies, only a small part has been implicated in the promotion of aging by interactions between different mechanisms interacting with each other. As a whole, there are more or less mutual influence and interaction between various systems and organs of the human body. Many mechanistic studies of aging, especially the interactions between various aging mechanisms, are not clear. These issues remain to be further investigated in depth. There is still a long way to go before it is truly used in clinical practice. The problem of social labor can be greatly optimized if major breakthroughs are made in aging of the musculoskeletal system. In the future, Through the early intervention of aging, we can help middle-aged and elderly people effectively delay the development of musculoskeletal diseases with OA as the main disease. Reduce the public health care pressure caused by aging.

## Author contributions

YC: Writing – original draft. ZH: Writing – original draft, Writing – review & editing. HC: Writing – original draft. HL: Writing – original draft. KW: Writing – original draft. JC: Writing – original draft. ZL: Writing – original draft. YX: Writing – original draft. YL: Writing – original draft. SZ: Writing – original draft. SW: Writing – original draft. XZ: Writing – original draft. SJ: Writing – review & editing.
